# Prognostic analysis of combined curative resection of the stomach and liver lesions in 30 gastric cancer patients with synchronous liver metastases

**DOI:** 10.1186/1471-2482-12-20

**Published:** 2012-10-12

**Authors:** Yan-Na Wang, Kun-Tang Shen, Jia-Qian Ling, Xiao-Dong Gao, Ying-Yong Hou, Xue-Fei Wang, Jing Qin, Yi-Hong Sun, Xin-Yu Qin

**Affiliations:** 1 Department of General Surgery, Zhongshan Hospital of Fudan University, No 180 Fenglin Road, Shanghai, 200032, China; 2 Department of Pathology, Zhongshan Hospital of Fudan University, No 180 Fenglin Road, Shanghai, 200032, China

**Keywords:** Gastric cancer, Liver metastases, Clinicopathological factors, Prognosis

## Abstract

**Background:**

Gastric cancer with synchronous liver metastasis remains a clinical treatment challenge. There has been a longstanding debate on the question whether surgical resection could be beneficial to long-term survival. This study is to investigate the effectiveness and prognostic factors of combined curative resection of the stomach and liver lesions in gastric cancer patients with synchronous liver metastases.

**Methods:**

A total of 30 patients who underwent simultaneous curative gastric and liver resection from March 2003 to April 2008 were analyzed retrospectively. Univariate and multivariate analyses were performed to select independent factors for survival.

**Results:**

The overall 1-, 2-, 3- and 5-year survival rates of 30 patients were 43.3%, 30.0%, 16.7% and 16.7%, respectively, with a median survival of 11.0 months and 5 patients still living by the time of last follow-up. Single liver metastasis (*p* = 0.028) and an absence of peritoneal dissemination (*p* = 0.007) were significantly independent prognostic factors for these gastric cancer patients with synchronous liver metastases. Major adverse events were protracted stomach paralysis in 2 patients and pulmonary infection in another 2 patients, all of whom recovered after conservative treatment.

**Conclusions:**

This descriptive study without control group found that patients with solitary liver metastasis and absence of peritoneal dissemination could have better survival benefit from simultaneous curative resection of the gastric cancer and liver metastases.

## Background

Liver is one of the most frequent sites of cancer metastasis from gastrointestinal origin, and the major cause of disease death from stomach cancer [1]. The 5-year survival rate could be up to 29% for metachronous liver metastasis and only 6% for synchronous liver metastasis, from gastric cancer [2]. Therefore, it has long been thought by many that surgical treatment could not bring any substantial survival benefit for gastric cancer patients with synchronous liver metastasis. However, there has also been longstanding debate on the question whether surgical resection could be beneficial to long-term survival. Some believe that if R0 resection could be performed for both gastric cancer and synchronous liver metastasis, such simultaneous resection could significantly improve survival [3,4]. On the other hand, gastric cancer patients with liver metastasis usually have multiple intrahepatic lesions, peritoneal metastasis, regional lymph nodes metastasis and adjacent organs involvements [5-7], making it questionable whether simultaneous resection could bring any survival benefit. From March 2003 to April 2008, we performed simultaneous resection of both the gastric cancer and liver metastasis on 30 patients. This study is to summarize our experience and to analyze the efficacy and prognosis on these patients.

## Methods

The study complied with the declaration of Helsinki and was approved by Biomedical Research Ethics Committee of Zhongshan Hospital of Fudan University (NO.2009-160). All subjects gave written informed consent.

### Patients and treatment

From March 2003 to April 2008, a total of 2942 patients with gastric cancer were treated at our institution. From the archived medical records, a complete database was established on 30 gastric cancer patients with synchronous liver metastasis, who had simultaneous complete resection of both gastric cancer and liver metastasis. The database covered all clinico-pathological characteristics. Lymph node grouping was based on Japanese classification on cancer typing [8] and TNM classification was base on AJCC 7th edition [9].

Of these 30 patients were 27 males and 3 females, with age ranging from 33 to 72 years old (median 60 yr). The primary stomach cancer was located at the antrum in 11 cases, at the gastric body in 9 cases and at the cardia-fundus region in 10 cases. On local invasion status, 4 cases had tumor invasion beneath the serosa, and the remaining 26 cases all had tumor invasion beyond the serosa. On lymph nodes status, 7 cases did not have lymph nodes metastasis while the remaining 23 cases were lymph nodes positive. In terms of liver metastasis, 22 patients had one intrahepatic metastasis lesion and 8 patients had 2–3 liver metastases. There were 27 patients with metastasis limited to one lobe of the liver (H1), and 3 cases with metastasis on both lobes of the liver (H2). Simultaneous peritoneal metastasis was found in 5 patients. Of surgical approaches, 11 patients had curative distal gastrectomy, 10 patients had curative proximal gastrectomy and 9 cases had total gastrectomy. For liver resection, 7 patients had lobectomy and 23 patients had partial hepatectomy. These patients did not receive any preoperative chemotherapy, but all patients had postoperative adjuvant chemotherapy by experienced oncologists.

### Follow-up

All patients were regularly followed-up by telephone, with the last follow-up on April 14, 2012. The survival was calculated from the date of surgery to the date of death or last follow-up. There were 25 cases died, all due to cancer recurrence.

### Statistical analysis

All the data were analyzed with SPSS 16.0 software. The survival was analyzed by Kaplan-Meier and log rank test. Cox regression model analysis was performed for univariate and multivariate analysis, so as to discover independent prognostic factors. Two-sided *p* < 0.05 was considered as statistically significant.

## Results

### Perioperative features

There were 14 (46.7%) cases with blood transfusion (300–1000 mL, median 520 mL), 24 (80.0%) cases with albumin transfusion (20–100 g, median 43 g), and 12 (40.0%) cases with parenteral nutrition support. Two (6.7%) patients had gastric paralysis after operation and subsequently recovered after nasogastric tube depression for 19 and 22 days, respectively. Two (6.7%) patients had pulmonary infection, one with A. baumanii and treated with antibiotics for 49 days, and the other one infected with Klebsiella pneumoniae and treated for 10 days with anti-infection agents, and both were recovered well. There were no perioperative deaths.

### Follow-up Results

**Figure 1:**
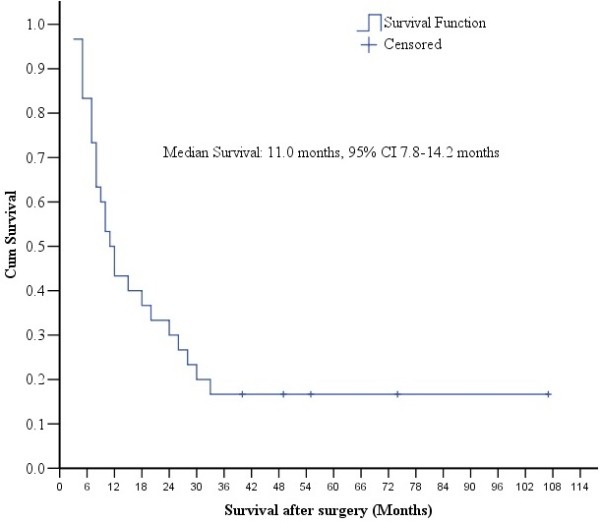
**Median survival in 30 patients.** Kaplan-Meier survival curve of the 30 patients in this study showed a median survival of 11.0 months after the simultaneous curative resection of the stomach and liver metastases.

All 30 patients had complete follow-up data. By the time of last follow-up, 25 (83.3%) patients died and 5 (16.7%) patients were living, with median survival of 11.0 months (95% CI 7.8 to 14.2 months). The 1-, 2-, 3- and 5-year survival rates were 43.3%, 30.0%, 16.7% and 16.7%, respectively (Figure 1). One patient with gastric ulcerative hepatoid adenocarcinoma (pT2N2M1) lived for 107 months without evidence of recurrence.

### Analysis on survival related independent factors

**Figure 2:**
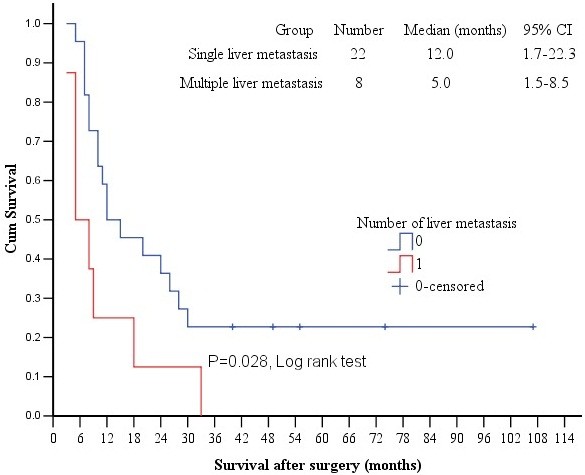
**Comparison of survivals in patients with single liver metastasis and with multiple liver metastases.** The Kaplan-Meier survival curves showed a significantly longer survival time in patients with single liver metastasis than those with multiple liver metastases.

**Figure 3:**
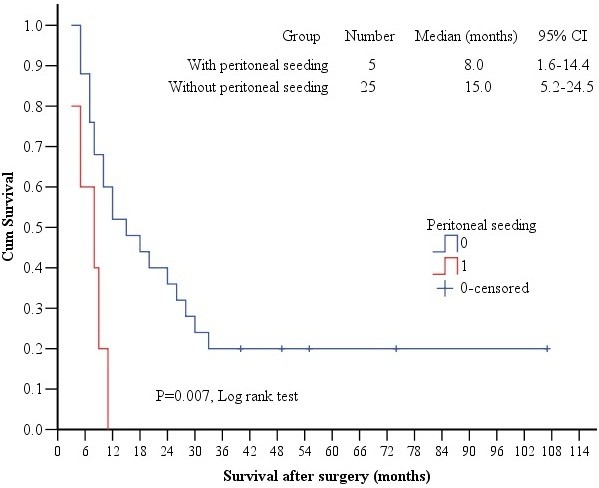
**Comparison of survivals in patients with versus without peritoneal metastasis.** The Kaplan-Meier survival curves showed a significantly shorter survival time in patients with versus without peritoneal metastasis.

Table 1 summarized the correlation of major clinico-pathological factors with survival status. Age, gender, tumor marker levels (CEA, AFP and CA19-9), primary tumor size, liver metastasis lesion size, tumor emboli, ascites, T staging and N staging all had no significant correlation with survival, but the number of liver metastatic lesions (log rant test, *p* = 0.028, Table 2, Figure 2) and peritoneal metastasis (log rant test, *p* = 0.007, Table 2, Figure 3) were significantly correlated with survival. Multivariate Cox regression survival analysis also confirmed that number of liver metastasis and peritoneal metastasis were independent prognostic factors (Table 3).

**Table 1:** Clinico-pathological features on 30 patients in this study

**Age (yr)**	
Median (Range)	60 (33—72)
Gender: n (%)	
Male	27 (90.0%)
Female	3 (10.0%)
Gastric cancer site: n (%)	
Gastric antrum	11 (36.7%)
Gastric body	9 (30.0%)
Gastric cardia-fundus	10 (33.3%)
Elevated tumor markers: n (%)	
CEA	7 (23.3%)
AFP	6 (20.0%)
CA19-9	8 (26.7%)
Gastric cancer diameter: cm	
Median (Range)	3.7 (1.0—11.0)
Pathological type: n (%)	
Papillary adenocarcinoma	13 (43.3%)
Tubular adenocarcinoma	10 (33.3%)
Poorly differentiated adenocarcinoma	4 (13.3%)
Mucinous adenocarcinoma	3 (10.0%)
Number of intrahepatic metastases: n (%)	
Single metastastic lesion	22 (73.3%)
Multiple metastatic lesions	8 (26.7%)
Diameter of intrahepatic metastasis lesions: cm	
Median (Range)	3.1 (0.5—16.0)
Surgical approaches: n (%)	
Distal gastrectomy	11 (36.7%)
Total gastrectomy	9 (30.0%)
Proximal gastrectomy	10 (33.3%)
T classification: n(%)	
T1	1 (3.3%)
T2	3 (10.0%)
T4a	26 (86.7%)
N classification: n (%)	
N0	7 (23.3%)
N1	3 (10.0%)
N2	5 (16.7%)
N3	15 (50.0%)
Peritoneal metastasis	
P0	25 (83.3%)
P1	5 (16.7%)
Tumor embolus	
Yes	13 (43.3%)
No	17 (56.7%)
Tumor differentiation: n (%)	
Well differentiated	2 (6.67%)
Intermediately differentiated	5 (16.7%)
Poorly differentiated	23 (76.7%)
Survival status: n (%)	
Survived	5 (16.7%)
Died	25 (83.3%)

**Table 2:** Survival analysis

**Items**	**N (survival rate)**	**Log rank*p***	**HR**	**95% CI**	***P***
Age: (yr)					
≤ 60 yr	15 (13.3%)	0.617	0.822	0.374-1.808	0.627
> 60 yr	15 (20.0%)				
Gender: n(%)					
Male	27 (18.5%)	0.725	0.809	0.238-2.748	0.734
Female	3 (0)				
CEA					
Normal	23 (13.0%)	0.499	0.716	0.265-1.936	0.497
Increased	7 (28.6%)				
AFP					
Normal	24 (16.7%)	0.728	1.185	0.443-3.172	0.736
Increased	6 (16.7%)				
CA19-9					
Normal	22 (13.6%)	0.527	0.748	0.297-1.886	0.538
Increased	8 (25.0%)				
Ascites					
Yes	7 (17.4%)	0.793	1.127	0.449-2.833	0.799
No	23 (14.3%)				
Primary tumor size					
< 5cm	18 (22.2%)	0.984	1.008	0.454-2.236	0.985
≥ 5cm	12 (8.3%)				
Number of liver metastasis					
Single	22 (22.2%)	*0.028	2.456	1.048-5.756	*0.039
Multiple	8 (8.3%)				
Liver metastasis size					
< 5cm	14 (21.4%)	0.766	1.124	0.509-2.482	0.772
≥ 5cm	16 (12.5%)				
Peritoneal metastasis					
P1	5 (20.0%)	*0.007	3.836	1.292-11.383	*0.015
P0	25 (0)				
Liver surgery					
Lobectomy	23 (17.4%)	0.944	1.032	0.411-2.593	0.946
Partial hepatotrectomy	7 (14.3%)				
T stage: n(%)					
T1, T2	4 (50.0%)	0.508	0.757	0.324-1.767	0.519
T3, T4	26 (11.5%)				
N metastasis: n(%)					
Negative	7 (14.3%)	0.574	1.293	0.515-3.249	0.584
Positive	23 (17.4%)				
Tumor embolus					
Yes	13 (15.4%)	0.650	1.196	0.541-2.647	0.658
No	17 (17.6%)				
Tumor differentiation					
Well-intermediately differentiation	7 (28.6%)	0.379	1.535	0.573-4.112	0.394
Poorly differentiated	23 (13.0%)				

**Table 3:** Multivariate Cox regression analysis

	**HR**	**95% CI**	**P value**
Peritoneal metastasis (P0 *vs* P1)	3.481	1.159-10.458	*0.026
Number of liver metastasis (1 *vs* 2–3)	2.262	1.056-5.349	*0.043

## Discussion and conclusions

Liver is one of the most frequent sites of cancer metastasis from gastrointestinal origin, and the major cause of disease death from stomach cancer. The incidence of synchronous liver metastasis from gastric cancer is about 2.0%-9.6%, which is lower than that from colorectal cancer. Approximately 0.4%-1.0% of these patients could be treated by liver resection [7,10-12], with median survival of 5–31 months, 1-year survival rate of 15%-77%, and 5-year survival rate of 0%-38%, after hepatectomy [6,7,13-17].

In the current study, 30 gastric cancer patients with synchronous liver metastasis were simultaneously treated by both gastrectomy and hepatectomy, resulting a median overall survival of 11.0 months, and 1-, 2-, 3- and 5-year survival rates of 43.3%, 30.0%, 16.7% and 16.7%, respectively. Of particular note, 1 patient has a disease-free survival of 107 months. Our multivariate analysis found that preoperative tumor marker levels, primary tumor size, tumor invasion depth, lymph nodes metastasis, histological types, tumor emboli, ascites, liver metastasis sizes all had no significant impact on survival, but the number of liver metastasis and peritoneal metastasis did have significant impact on survival.

It has been reported that gastric cancer prognosis could be heavily influenced by many tumor pathological features such as tumor invasion depth, lymph nodes metastasis, pathological types and tumor emboli [2,7,16,18].This study, however, did not find any significant survival impact of these features, most probably due to the fact that most previous studies included patients with both synchronous and metachronous metastases, but our study only focused on gastric cancer patients with synchronous liver metastasis. Many other studies since 2001 [2,3,6,13,16,19] also suggested that pathological staging of the primary tumor did not have significant impact on postoperative survival. Based on these results, we believe that the routine clinico-pathological features of the primary gastric cancer are not major factors to impact on postoperative survival in such patients with simultaneous resection of the gastric cancer the liver metastasis.

In our study, we found that the number of liver metastases and peritoneal metastasis are independent prognostic factors for such patients with simultaneous resection. Okano et al. [11] also found that patients with single liver metastasis had significantly higher 3-year survival rate than those with multiple liver metastases. In addition, several other reports [6,16,20] also confirmed that the number of liver metastases is a major prognostic factor. Ueda et al. [21] also found in 72 gastric cancer patients with liver metastasis who had simultaneous resection, that patients with H1 and no peritoneal metastasis had better survival, and such result was repeated in a another similar study [19]. Because the number of liver cancer metastases had strong correlation with distribution of liver metastases (one lobe or two lobes), the prognostic significance of liver cancer metastasis distribution should be further investigated in large scale clinical studies.

This study did not find any independent survival impact of conventional pathological factors such as T stage and N stage. Two major reasons may account for such difference. The first concerns the disease status in our series. This study included 30 patients of gastric cancer with synchronous liver metastasis. In addition, there were also 5 patients with peritoneal metastasis. Therefore, all these patients were clinical stage IV. So it is not surprising that the multivariate analysis found that the number of liver metastases and peritoneal metastases were the only two independent factors influencing survival. The second concerns the number of patients at different T and N stages in this study. There were only 4 T1 and T2 cases, and 26 T4a cases. Similarly, there were only 10 N0 and N1 cases out of the total 30 cases. The smaller the number, the less statistical power they had. The much smaller number of early T and early N cases may also account for reason why they seemed not to have influence on overall survival after multivariate analysis.

Among the 30 cases in this cohort, 6 patients had increased AFP levels. These patients may had gastric hepatoid carcinoma, which is a special subtype of gastric cancer having very aggressive evolution. As the number was not large enough, it is not possible to reach definite conclusions on such patients. Accumulation of more patients is warranted to make a more comprehensive study of this patient subpopulation.

To our knowledge, our study is the largest series from China to report on the simultaneous resection of gastric cancer and liver metastasis. Our conclusion is that patients with single liver metastasis and no peritoneal metastasis could have better prognosis after simultaneous resection of both lesions. Although this was a retrospective observational study without control group, the results could be helpful to form rational treatment approaches for such patients in China.

Liver metastasis is not the absolute contraindication for gastric cancer surgery, but the following conditions should be considered in selecting patients. First, the primary tumor could be resectable, and there should be no superclavicular lymph nodes metastases or abnominal aorta lymph nodes metastasis, no extrahepatic metastasis or peritoneal metastasis. Secondly, there exists single liver metastatic lesion, or lesions confined to one lobe of the liver. Thirdly, the patient should have good organ function reserve, with basically normal cardiac, pulmonary, hepatic and renal functions. Absolute contraindications are extrahepatic metastases and unresectable liver metastases. Whether preoperative chemotherapy could be helpful to reduce liver metastasis or to enhance the possibility of a clean margin resection, we did not conduct any study on this point. Future work should consider this option.

Based on our results and literature study, we concluded the for gastric cancer patients with liver metastasis: (1) careful preoperative evaluation should be performed to consider if curative resection is possible, and laparoscopy could be considered if necessary; (2) for patients with synchronous single liver metastasis, curative resection should be the treatment of choice; (3) patients with peritoneal metastasis had poor survival; (4) preoperative tumor marker levels should not be the criteria to judge whether surgery should be performed; and (5) the pathological staging of the primary tumor does on have significant impact on postoperative survival.

## Competing interests

The authors declare that they have no competing interests.

## Authors’ contributions

KTS, XYQ, YHS and JQ designed research; YNW, JQL, XDG and XFW conducted research; YYH provided pathological databases; YNW and XFW analyzed data or performed statistical analysis; YNW and KTS wrote paper; KTS had primary responsibility for final content; All authors read and approved the final manuscript.

## Pre-publication history

The pre-publication history for this paper can be accessed here:

http://www.biomedcentral.com/1471-2482/12/20/prepub
